# Gas Sensors Based on Semiconducting Metal Oxide One-Dimensional Nanostructures

**DOI:** 10.3390/s91209903

**Published:** 2009-12-04

**Authors:** Jin Huang, Qing Wan

**Affiliations:** Key Laboratory for Micro-Nano Optoelectronic Devices of the Ministry of Education and State Key Laboratory of Chemo/Biosensing and Chemometrics, Hunan University, Changsha, 410082, China; E-Mail: huangjin8607@gmail.com

**Keywords:** gas sensors, semiconducting oxides, one-dimensional nanostructures

## Abstract

This article provides a comprehensive review of recent (2008 and 2009) progress in gas sensors based on semiconducting metal oxide one-dimensional (1D) nanostructures. During last few years, gas sensors based on semiconducting oxide 1D nanostructures have been widely investigated. Additionally, modified or doped oxide nanowires/nanobelts have also been synthesized and used for gas sensor applications. Moreover, novel device structures such as electronic noses and low power consumption self-heated gas sensors have been invented and their gas sensing performance has also been evaluated. Finally, we also point out some challenges for future investigation and practical application.

## 1. Introduction

Semiconducting metal oxides have been known for decades to be good gas sensing materials. Ethanol sensors based on SnO_2_ thick films have been commercialized for years. In 1991, Yamazoe demonstrated that reduction in crystal size would significantly increase the sensor performance [1]. This is because nanosized grains of metal oxides are almost depleted of carriers (most carriers are trapped in surface states) and exhibit much poorer conductivity than microsized grains in ambient air, hence, when exposed to target gases, they exhibit greater conductance changes as more carriers are activated from their trapped states to the conduction band than with microsized grains. Thus, the technological challenge moved to the fabrication of materials with nanocrystals which maintained their stability over long-term operation at high temperature [2]. The exploration of one-dimensional (1D) oxide nanostructures has been stimulated and facilitated by the convenience of obtaining large amounts of single crystalline nanowires/nanobelts via the vapor transport [3] and vapor-liquid-solid (VLS) methods [4]. The Sberveglieri [5] and Yang [6] groups initiated the investigation of gas sensing properties of SnO_2_ nanobelts. Sberveglieri *et al.* demonstrated the use of SnO_2_ nanowires as sensor materials showing prominent current changes towards ethanol and CO, respectively, in a synthetic air environment [5], while Yang *et al.* demonstrated the first photochemical NO_2_ nanosensors (based on individual SnO_2_ nanoribbons) operating at room temperature [6]. In 2004, our group reported high-performance ZnO nanowire sensors with a low detection limit of 1 ppm ethanol at 300 °C [7]. Ever since then the number of reports on gas sensors based on 1D metal oxide nanostructures have been growing exponentially every year.

The article presents a comprehensive perspective on research efforts made in gas sensor fabrication and testing based on one-dimensional metal oxide nanostructures in recent years, during which electrospun oxide nanofibers have been gaining attention in gas sensor applications [8-15]. The data on film type nanosensors reviewed in this article are mainly published in 2008 and 2009. In Section 2, gas sensor configurations and measurements, performance parameters, as well as theoretical fundamentals of gas sensors based on 1D nanostructures will be introduced, while the material system, cited are focused on undoped metal oxide nanowires or nanobelts. Section 3 features modified 1D metal oxide nanostructures, as well as heterostuctures. In Section 4, novel gas sensors based on novel operation principles such as the “electronic nose”, the self-heated gas sensor and the optical gas sensor will be elaborated on. Finally, Section 5 summarizes the whole article and indicates possible future developments in one-dimensional metal oxide nanosensors.

## 2. Fundamentals of Gas Sensors Based on Metal Oxide 1D Nanostructures

### 2.1. Fabrication and Characterization of Gas Sensors

**Figure 1.**
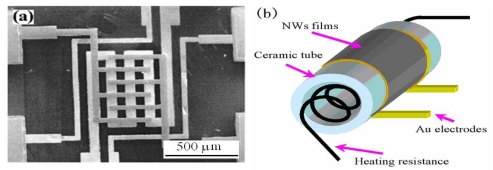
(a) MEMS structures with interdigitated electrode [7]. (b) Schematics of nanowire gas sensors on ceramic tube [26].

**Figure 2.**
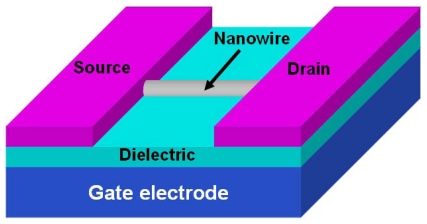
The schematic of the single nanowire field effect transistor.

Up to now, one-dimensional metal oxide nanostructures sensors have been characterized in three ways: conductometric, field effect transistor (FET) and impedometric ones. Conductometric sensors are based on resistance changes caused by exposure of the sensor surface to a target species. So far, two types of conductometric nanowire gas sensors have been mainly fabricated: one is the film type, in which a film composed of nanowires is contacted by pairs of metal electrodes on a substrate (Figure 1a) or a ceramic tube (Figure 1b); the other is the single nanowire type in which a single nanowire bridges two metal electrodes on a heavily doped silicon substrate covered with SiO_2_ acting as insulating layer between the nanowire/electrode combinations and the conducting silicon (Figure 2). In the fabrication of film type nanosensors, nanowires products are usually pulverized to a pulp state and either directly painted or screen-painted [16] onto the substrates or tubes. But other approaches are reported. Sometimes nanowire growth is integrated into device fabrication [17-20]: SiO_2_/Si substrates with patterned metal coatings were used to catalyze the growth of the metal oxide nanowires and the coating also acts as electrodes contacting the sensing material. This type of sensor has lower contact resistance compared to the previous one because the nanowire growth process is integrated into device fabrication. Well-aligned nanowire arrays have been fabricated into nanosensors to explore benefits brought about by orderliness.

**Figure 3.**
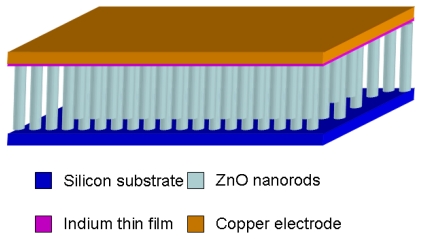
The schematic of the ZnO nanorod array sensor.

**Figure 4.**
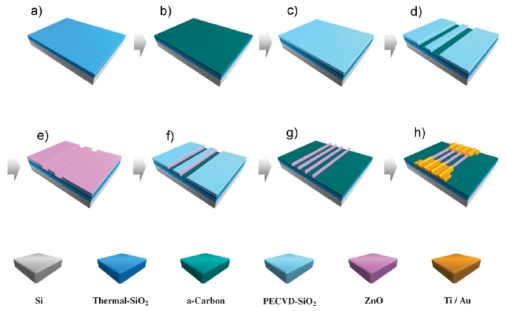
Schematic diagram illustrating the fabrication processes of the ZnO nanowire device based on nanoscale spacer lithography (NSL): (a) thermal growth of SiO_2_ layer, (b) deposition of a-carbon thin film (act as etch-stop layer in subsequent process), (c) plasmon enhanced chemical vapor position (PECVD) of SiO_2_ thin film (sacrificial layer), (d) sacrificial layer patterning, (e) atomic layer eposition (ALD) of ZnO thin film, (f) top view of the chip after plasma etching of ZnO, (g) sacrificial layermoval,and (h) top view of the ZnO nanowire device after metal electrode deposition [24].

For instance, Figure 3 shows the sensor device structure that has been adopted in exploring gas sensing properties of ZnO nanorods [21], in which ZnO nanorod arrays are sandwiched between the silicon substrate supporting their growth and an indium thin film that forms ohmic contact with the nanorods and the copper electrode. Another interesting approach to assemble nanosensors involves combining two vertically aligned CuO nanowire arrays into a three-dimensional nanostructure, in which two pieces of nanowire arrays were attached to the copper plate and the micromanipulator tip (copper wire) and the distance between the two arrays can be adjusted. The sensor built in this style was reported to be capable of detecting air-diluted H_2_S at the parts per billion level [22]. However, these aligned nanowire arrays are grown by “bottom up” methods, and the orderliness is not as good as those fabricated via “top to bottom” approaches. Francioso and Son *et al.* employed microelectronics processes such as photolithography and plasma etching (demonstrated in Figure 4) to fabricate TiO_2_ [23] and ZnO [24] parallell nanowire arrays, respectively, and investigated their gas sensor behaviors. Son *et al.* [25] developed an alternative technique to construct well-aligned nanowire arrays, which takes advantage of the two facts: 1) the step edges of terraces are energetically favorable for the nucleation of adatoms; 2) a low deposition rate is favorable for producing localized nucleation at the step edges of terraces. They prepared uniform terraces on (0001) sapphire substrates by annealing a miscut sapphire substrate and minimized the ZnO deposition rate by using low laser pulse repetition rate as well as a shadow mask which blocks direct ZnO plume generated by laser ablation pulsed laser deposition (PLD).

Basically, the working principle of a FET type sensor is that the species adsorbed onto the channel surface can work as an extra virtual gate bias and hence cause changes in the apparent threshold voltage. However, the FET configuration of a sensor does not guarantee it work in the simple ideal way. For example, Andrei *et al.* [27] discovered that their SnO_2_ nanobelt FET can be modeled as two Schottky diodes connected back-to-back with a series resistance from the nanobelt separating the diodes and only work as a FET in the presence of hydrogen. Another interesting phenomenon observed by Zhang *et al.* is that the gate effect typical of a FET was substantially weakened when their In_2_O_3_ nanowire transistors were exposed to high concentrations of NH_3_ (10%) [28]. They proposed that these NH_3_ molecules residing on the nanowire surface can be charged and discharged by sweeping the gate bias and hence effectively work as charge traps screening the electric field induced by the gate bias.

Impedometric sensors are based on impedance changes and are operated under alternating voltage upon exposure to target species. Like conductometric sensors, there are two types of impedometric ones: film type [29] and single nanowire type [30]. But this group of sensors has not attracted as much attention as the conductometric sensor yet. The rest of Section 2 will be mainly focused on conductometric sensors.

### 2.2. Surface Reactions and Models for Gas Sensors

Semiconducting oxides generally owe their conductivity to their deviation from stoichiometry. Defects such as interstitial cation or anion vacancies also play an important role in their conductivity. Target species can be classified into two groups: oxidizing gases or electron acceptors such as NO_2_, which produce a decrease in the conductance of n-type semiconducting materials (*i.e.*, electrons are the major carriers, such as ZnO, SnO_2_, In_2_O_3_) and an increase in the conductance of p-type semiconducting materials (*i.e.* holes are the major carriers, such as CuO); reducing gases or electron donors such as H_2_S, CO, H_2_ and water vapor, which act in a reverse manner. Interestingly, sensors based on TiO_2_ nanofibers have recently been reported not to undergo conductance decrease towards oxidizing gas NO_2_ as they normally do [31].

**Figure 5.**
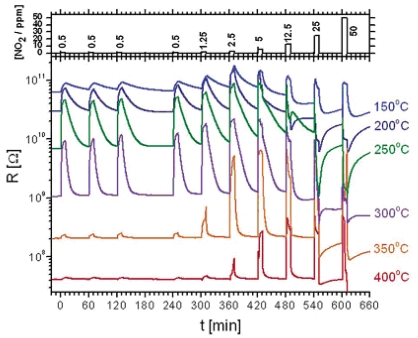
The resistance response during cyclic exposure to 10 min pulses with increasing concentrations of NO_2_ mixed in dry air at various operating temperatures [31].

As shown in Figure 5, at higher concentration ranges the device resistance plummeted before NO_2_ stimulus was removed. The authors attributed such an abnormal response behavior to the conduction type inversion (n-to-p) of the sensing material whose conduction is surface-trap limited, owing to the high surface-to-volume ratio of this material.

There are two types of adsorption: physisorption, the first step of the association of the gas species with the sensor surface, and chemisorptions, which involves exchange of electrons between the adsorbed species and the material surface. The major difference between these two processes is that physisorption is exothermic while chemisorption is endothermic, precisely an activated process whose activation energy can be supplied by thermal or non-equilibrium ones such as illumination [2]. This leads to the fact that physisorption predominates in low temperature range whereas chemisorption dominates in higher temperature range. The sensing characteristics of metal oxides are widely considered to be related with chemisorbed oxygen and water, which can act as intermediates catalyzing the charge transfer processes between gas species and the bulk and which complicates the study of gas sensing mechanisms. The major way they interfere with the gas sensing process is through fluctuations in the concentration and the charges of oxygen vacancies. Ahn *et al.* [32] investigated the effect of oxygen-vacancy-related defects on gas-sensing properties of their single ZnO nanowire gas sensors and found that the gas sensitivity towards NO_2_ was linearly proportional to the photoluminescence intensity of oxygen-vacancy-related defects. Their work proves evidence of the role that oxygen vacancies play in gas sensing.

Recent development in the synthesis of single crystalline nanowires or nanobelts has stimulated research into their gas sensing properties, which reveal important information about the reactions between target species and metal oxide surfaces free from complications caused by grain boundaries. For example, single crystalline SnO_2_ nanobelts provided Moskovits *et al.* the opportunity to study surface reaction kinetics between the individual nanobelt surface and CO and fit the experimental data to the analytical model they derived [33]. Another example is that single crystalline SnO_2_ nanobelts with well-defined facets [with exposed (1 0 1) and (0 1 0) surfaces] also give Yang *et al.* the model to verify their results from numerical investigation into surface interactions between SnO_2_ with NO_2_ species: through first-principle density functional theory (DFT) calculations. They found unexpectedly that most stable adsorbed species involve an unexpected NO_3_ group doubly bonded to Sn centers, which was confirmed by their X-ray absorption spectroscopy studies on nanoribbons [34].

Nanowire/nanobelt diameter is usually on the order of several nanometers and is comparable to the Debye length and this often results in much larger sensitivity than their thin film or bulk counterparts. The size dependent characteristic has also been studied by some researchers. Liao *et al.* found that thin nanorods have a significantly better sensing performance than thick nanorods in the detection of C_2_H_5_OH and H_2_S (100 ppm) in air [21]. The gas performance of film type gas sensors can be limited not just by surface reaction processes, but also by the morphology and microstructure of the films. Contact barriers among nanowires can also affect the gas sensing properties via affecting the resistance of the bulk material [35]. Generally, researchers use the power law, S = a + bc^ρ^, to fit the concentration-sensitivity curves of film type nanosensors, and Langmuir adsorption isotherm to fit the sensitivity-concentration curves of single nanowire gas sensors [36].

### 2.3. Performance Parameters

Sensitivity, response and recovery time, linear range, as well as limit of detection (LOD) are important performance parameters for gas sensors. The sensitivity of conductometric sensors is defined as the ratio of the device's resistance when exposed to target species to that in ambient air, exactly R_g_/R_a_ (where R represents resistance, the subscript ‘g’ represents target gas, and ‘a’ represents ambient air) if the target gas is an oxidizing one, or G_g_/G_a_ (G represents conductance) if it is a reductive one. Response (recovery) time is defined as the time period needed for the device to undergo resistance changing from 10% (90%) to 90% (10%) of the value in equilibrium upon exposure to an oxidizing (reducing) analyte. According to its definition, the estimation of LOD is done via extrapolating the R_g_/R_a_ versus concentration curve to 3σ/R_a_ (σ is the standard deviation of R_a_), but very few references mention to have done it in this way [31,37]. This is mainly because of the morphological complexity of the porous sensor surface and lack of efficient model to fit the sensitivity-concentration curves.

There are few problems in this field of study which can severely hinder real applications of metal oxide 1D nanostructures. First, the researchers in this community do not abide by a unified LOD when claiming the detection limit of their gas sensor reaches some ppb or ppm level. Generally they just label the lowest concentration of the analyte used in their test as the detection limit of their gas sensors. The second is lack of uniformity in the working temperature selected. Almost half the publications report a working temperature setting of 300 °C, and one quarter at 400 °C. The optimum working temperature is not always explored. Third, as water vapor produces resistance changes for metal oxides, it is important to present humidity information. Fourth, very few researchers (except [30,38-40]) have worked outside the linear range or selectivity to facilitate industrial applications.

Up to now, various metal oxides 1D nanostructures (SnO_2_ nanowhiskers, In_2_O_3_ nanowires, ZnO nanorods, WO_3_ nanowires, TeO_2_ nanowires, CuO nanoribbons, CdO nanowires *etc.*) have been fabricated into film type nanosensors. As shown in Table 1, the most widely studied substances are SnO_2_ and ZnO, probably due to the convenience of obtaining large quantities of SnO_2_[7] or ZnO nanowires [41] via thermal evaporation or a vapor-liquid-solid method. Actually this Table serves as a supplementary to another one in reference [42], which provides additional information on reported gas sensor properties. It's noteworthy that, in agreement with intuition, gas sensitivities of single nanowire gas sensors are invariably far less than those of nanowire film gas sensors, but the significance of single nanowire gas sensors is their potential application in microarray electronic noses [43].

The metrics of humidity senor involves more complicated procedures than sensors for other target species in order to obtain reliable data. In the latter, the ambient gas is usually switched between air and a target gas diluted in air, which simulates real applications; in the former, the target gas (water vapor) has to be injected into a highly dry environment which requires pretreatment such as evacuation to remove water adsorbent in the chamber [44]. When testing is conducted under high vacuum, the usual concept, the concentration, used to represent the quantity of gas species present is replaced by an alternative one, the gas pressure of target species.

**Figure 6.**
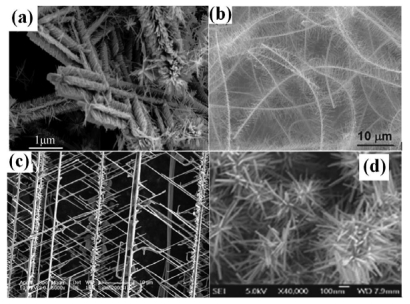
(a) SEM images of ZnO brushes [67]. (b) SnO_2_ brushes [28]. (c) ZnO dendrites [68]. (d) ZnO nanoflowers [69].

Hierarchical, dendritic, or branched nanostructures (Figure 6) are potential benefits brought about by their unique morphology that have also been explored. Table 2 shows the performance of gas sensors made from such nanostructures. Here we should point out that unlike the other three types of structures which are homogeneous, SnO_2_ nanobrushes are composed of heavily doped back-bone nanowires, which exhibit metallic conduction and provide more electron paths in the three-dimensional porous film, and semiconducting undoped SnO_2_ nanowires which act as efficient sensing components.

Hundreds of publications have reported on the gas sensor behaviors of metal oxide 1D nanostructures since 2002. Since the lowest detection concentration is a very important performance index for sensors, it is important to make a summary on the level achieved towards common target species (CO, NO_2_, NH_3_, ethanol, H_2_, H_2_S) of sensors fabricated from different metal oxide 1D nanostructures. Table 3 is the result of such effort. This can be referred by those who wish to publish new data in this realm.

## 3. Modified Nanowires and Heterostructures

### 3.1. Modified Nanowires

**Figure 7.**
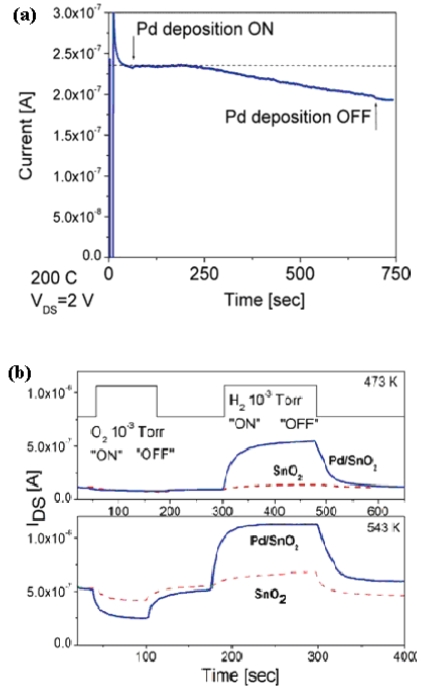
(a) changes in the source drain current during the early stage of Pd deposition onto a SnO_2_ nanobelt. (b) current response of an undoped (dashed line) and Pd-functionalized (solid line) nanostructure to sequential oxygen and hydrogen pulses at 473 K (right top pane) and 543 K (right bottom) [72].

It is widely accepted that the presence of noble metal elements (Pt, Pd, Au, *etc.*) on the surface of a metal oxide enhances the interaction of reducing gases with the absorbed oxygen on the surface, hence modified oxide nanowires have also attracted some research attention. In 2002, Arbiol *et al.* reported the synthesis and structural study of Pd nanoparticles on SnO_2_ nanowhiskers and believed such system had enhanced sensor performance [71]. It was not until 2005 that such systems began to arouse attention for sensor fabrication and measurement (Figure 7). Kolmakov *et al.* [72] performed in-situ deposition of Pd nanoparticles in the same reaction chamber where the gas sensing measurements were carried out to ensure that the observed behavioral alteration was due to the Pd functionalization rather than the properties variation from one nanowire to another.

Figure 7a shows the conductance changing during the whole deposition process, in which Schottky barrier-type junctions resulted in the formation of electron depletion regions within the nanowire and constricted the effective conduction channel. Their Pd-functionalized SnO_2_ nanostructures exhibited a dramatic improvement in sensitivity toward oxygen and hydrogen, compared to pristine SnO_2_ nanostructures, due to the enhanced catalytic dissociation of the molecular adsorbate on the Pd nanoparticle surfaces and the subsequent diffusion of the resultant atomic species to the oxide surface (Figure 7b) [73].

Wang *et al.*, instead, studied the performance of sensor based on collective Pd nanocluster (sputtered) modified multiple ZnO nanorods and also found them to have enhanced sensitivity in detecting hydrogen: changes in room-temperature resistance of approximately a factor of 5 larger than unmodified ZnO nanorods upon exposure to hydrogen concentrations in N_2_ of 10–500 ppm [74].

**Figure 8.**
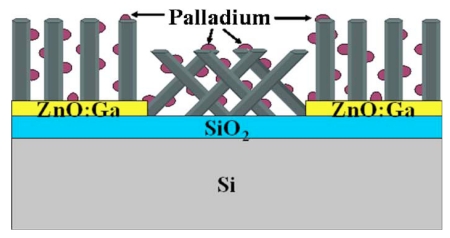
Schematic for the the cross sectional view of ZnO nanowire array deposited with Pd nanoparticles [75].

Chang *et al.* [75-77] employed a chemical-physical approach to decorate the nanowire surface with noble metal nanoparticles for film type nanosensor application: 1) immerse the nanowires sample in salt solution (PdCl_2_, HAuCl_4_); 2) place the sample under UV illumination for a period of time. During the illumination period the noble metal cations adsorb onto the nanowire surface and are reduced into atoms (from interaction with incident high energy photons) which coalesce and grow into nanoparticles. Moreover they used a patterned substrate to induce the growth of ZnO nanowires so that the decorated nanowires can self assembled into a nanofilm sensor (Figure 8). No further pulverization or grinding was involved as in standard film type nanosensor fabrication that may disintegrate the nanoparticle-nanowire structure. The thus-fabricated device exhibited enhanced response towards ethanol compared to non-decorated ZnO nanofilm sensors: increased from 18.5% to 44.5% at 170 °C and from 36.0% to 61.5% at 230 °C.

Pd-functionalized SnO_2_ nanowire film type gas sensors have also been fabricated and shown to have a sensitivity of 253 towards 2000 ppm H_2_ gas at 100 °C [78]. Gas sensors based on Pd nanocrystal-modified CeO_2_ nanowires were shown to be more sensitive than unmodified CeO_2_ nanowires towards CO; moreover, its response to CO is highly selective among other species such as H_2_, ethanol, gasoline, and H_2_S [79].

Doping is another technique utilized to improve gas sensing properties of metal oxides, where the dopant atoms are believed to act as activators for surface reactions. Ru-doped SnO_2_ nanowires were synthesized and demonstrated to undergo conductance changes upon exposure to liquefied petroleum gas (LPG), which is seldom used as target gas for measuring the gas sensing properties of SnO_2_ nanowires [80]. Our group fabricated film type nanowire sensors based on Sb-doped SnO_2_ single crystalline nanowires and found that they exhibited faster response and recovery compared to sensors based on undoped SnO_2_ nanowires[81].

### 3.2. Metal Oxide 1D Heterostructures

Heterostructures based on metal oxide nanostructures has also been synthesized and fabricated into gas sensors. Similar to those of noble metal decorated nanowires, an accepted mechanism of such systems is that the target gas molecules chemisorbs onto the hetero-interface which induces charge transferring process and modulates the barrier height and hence produce apparent resistance changing. Table 4 displays research efforts made in this aspect.

## 4. Novel Gas Sensors

Several prototypes of novel gas sensors operating under different physical and even statistical principles have been fabricated. One is based on existent technology, the basic idea of which is to make use of the fact that a metal oxide has different calibration curves (sensitivity-concentration relationship) or temperature characteristics (sensitivity-temperature relationship under specific concentration) towards different chemical species. Another makes use of the Joule heat produced by the sensor itself to achieve the optimum temperature rather than an external heater and hence reduce the total power consumption. The third one is based on a novel phenomenon that correlates the photoluminescence quenching effect metal oxides exhibit when exposed to NO_2_, whose mechanism remain unresolved.

### 4.1. Microarray Electronic Nose

The idea of “electronic noses” is inspired by the olfactory systems of humans and mammals. It discriminates different gas species by discerning the conductivity pattern of its various elements produced upon exposure to these gas species and hence is strongly dependent on data analysis and model building-up.

The most well-known way to fabricate a highly selective “electronic nose” is to integrate different recognition molecules on the same chip. However, this has been proven to not work well with metal oxides probably because the reactions do not vary much between a specific target species and different metal oxide surfaces. Chen *et al.* demonstrated a hybrid “electronic nose” composed of In_2_O_3_, ZnO, SnO_2_ nanowires and single-walled carbon nanotubes combined with statistical method principal component analysis (PCA) which cannot effectively distinguish H_2_ and ethanol molecules [88]. A general approach for fabricating metal oxides “electronic nose” is to separate one monolithic metal oxide thin film into sensor segments by parallel electrode strips used for measuring the conductance of the segments while a gas-permeable membrane layer with varying thickness deposited on top of the segments, along with a temperature gradient maintained along the segment arrays, forming two mechanisms to modify the selectivity of different segments [89].

**Figure 9.**
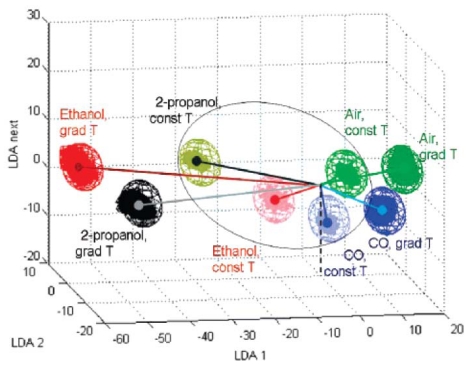
LDA analysis of the conductivity patterns obtained with SnO_2_ nanowire-based gradient microarray responses to different gas species (ethanol, 2-propanol and CO in the 2-10 ppm concentration range). The classification spheres correspond to normal distribution of data at 99.99% confidence level. The microarray operates under (a) quasihomogeneous heating at 580 K (const T areas inside the ellipse with dimmed colors) and (b) temperature gradient at 520-600 K (grad T areas with bright colors) [90].

The state-of-the-art multielectrode KAMINA platform is based on the second idea. Kolmakov *et al.* combined KAMINA technology with “bottom-up” SnO_2_ nanowire mats as the sensing elements [90]. They applied the statistical method linear discriminant analysis (LDA) to transfer sensor signals collected from all 38 channels into an optimized coordinate system of lower dimensionality equal to the number of training gases minus one (Figure 9).

The more different (separated) the conductivity patterns for the various trained gases, the better the discrimination power of the e-nose. Two points can be drawn from the Figure: (1) The isothermal (taken without thermal gradient) conductivity patterns of the nanowire sensor array are already sufficient to obtain substantially different signal patterns for the various gases; (2) If a temperature gradient is additionally applied along the microarray, the discrimination power is significantly enhanced. Their results suggested that the nanowire density variation over the segments alone can provide substantial differences in the conductivity patterns of the gradient microarray.

### 4.2. Self-heated Gas Sensors

**Figure 10.**
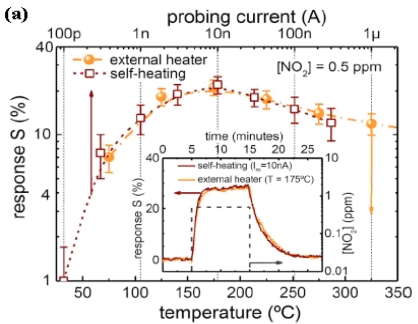

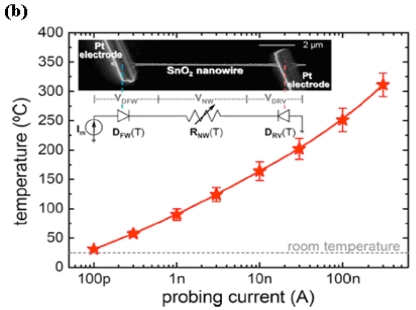
(a) comparison of the responses obtained with both measuring methodologies. The similarities to the calibration-curve of the external heater were used to roughly estimate the effective temperature obtained by the nanowire due to self-heating. The maximum response to this gas with and without heater (I_m_ = 10 nA) is the absolute equivalent (inset). (b) estimated temperature of the devices at different I_m_.

Kolmakov *et al.* [91] investigated the self-heating effect on the operation of single SnO_2_ nanowire gas sensors towards oxygen or hydrogen in oxygen and their sensor was able to operate without a heater, consuming only a few microwatts of power. Prades *et al.* [92] further tested the idea of optimizing the sensing conditions for the detection of gaseous species without the aid of an extra heater, which helps reducing the power consumption of the devices. They fabricated individual SnO_2_ nanowire devices and measured their sensing properties towards a specific concentration of NO_2_ under various input current and heating temperature respectively. Then they compared the calibration curve of each measuring method (Figure 10a) and generate a curve (Figure 10b) that correlate the input current and the effective temperature (roughly estimated through the response and recovery times obtained under each operating conditions) it achieves which can help further improvement of future prototype performances. Their devices operated optimally with less than 20μW for NO_2_ sensing to both bias and self-heating, and is significantly lower than the 140 mW required for the external microheater.

### 4.3. Optical Gas Sensing of NO_2_

**Figure 11.**
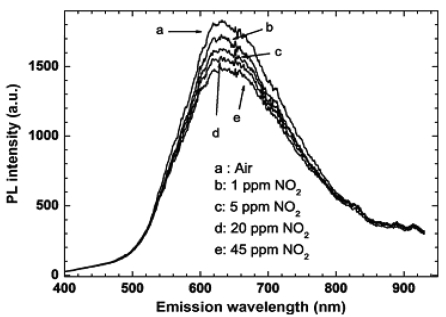
photoluminescence spectrum of SnO_2_ nanowires sample measured at room temperature in dry air and in nitrogen dioxide atmosphere at different concentrations [96].

Faglia *et al.* observed such phenomenon in SnO_2_ and ZnO nanowires: exposure to a few ppm concentrations of nitrogen dioxide significantly quenched the visible photoluminescence (PL) emission of nanowires (Figure 11), which was not affected by variation of relative humidity and by other interfering gases [93-95].

**Figure 12.**
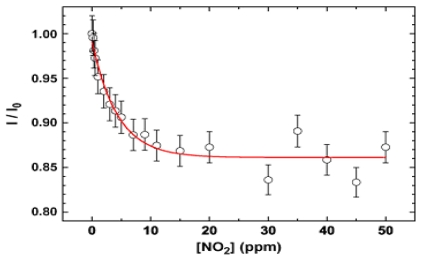
Quenching ratio at room temperature as a function of the NO_2_ concentration; solid curve is a best fit to data obtained by using the function I = I_B_ + (I_0_−I_B_)exp(−γ [NO_2_]). The I_B_ term represents a quencher-independent “background” contribution to PL [96].

The response is highly selective toward humidity and other polluting species, such as CO and NH_3_. They believed the results foresaw the development of a class of selective metal oxide gas sensors and pushed the lowest detection concentration of this novel configuration to 1ppm of nitrogen dioxide. Figure 12 can be viewed as the calibration curve of such optical gas sensor, whose sensitivity is defined as the ratio of PL intensity of SnO_2_ nanowires exposed to target gas (NO_2_) to the PL intensity of SnO_2_ nanowires exposed to blank sample (dry air).

In order to resolve the mechanism beneath, they studied the recombination dynamics based on time-resolved photoluminescence spectroscopy and numerical regression of the data by means of different decay functions (exponential, stretched exponential and double-exponential). Their findings suggest that adsorption of NO_2_ acts in de-activating the emitting transitions the same manner as in the static quenching mechanisms holding in bimolecular interactions [96].

## 5. Summary

This article has reviewed recent progress in the past two years in gas sensors based on semiconducting metal oxide one-dimensional nanostructures. During the past two years, although metal oxide nanowires/nanobelts have been exploited for detecting biological species such as glucose [97], urea [99], and L-tyrosine [100], or functioning merely as transducers in FET biosensors [101], numerous new data on their sensor properties towards common target species such as NO_2_, ethanol, H_2_, H_2_S has been published. Moreover, more modified or doped metal oxide nanowires/nanobelts have also been synthesized and their sensor properties towards these chemical gases investigated. Additionally, new device structures such as the “electronic nose” and the low power consumption self-heated gas sensor have been designed and their sensor response has also been evaluated. In the review, we have pointed out the lack of professionalism in many sensor performance measurements in several aspects. Specifically, there is the abuse of the concept of LOD, widespread failure to label the linear range or to assess the selectivity of the reported film type nanosensors, which make the results of little technological significance. Future development of this field would hopefully witness improvements in these two aspects.

## Figures and Tables

**Table 1.** Gas sensing properties of chemoresistors based on the one-dimensional nanostructures of different metal oxide.

**Materials**		**Target Gas**	**Lowest Detection Concentration**	**Response/Recovery**	**Ref.**

SnO_2_	Nano-whiskers	Ethanol	50 ppm (300 °C, S = 23)	N/A*/10 min	[46]
		H_2_	10 ppm (300, S = 0.4)	N/A	[47]

	Single nanowire	H_2_	100 ppm (2, S∼13)	N/A	[47]
		humidity	RH: 30% (30 °C, S∼1.25)	120-170 s/20-60 s	[48]

	nanorods	H_2_	100 ppm (150 °C)	N/A	[49]

In_2_O_3_	nanowires	Ethanol	100 ppm (370 °C, S∼2)	10 s/∼20 s	[50]
		NO_2_	1 ppm (250 °C, S∼2.57)	N/A	[51]
		H_2_S	200 ppb (RT*)	2–3 min/N/A	[52]
		Ethanol	5 ppm (330 °C, S∼1.84)	6 s/11 s	[53]

	Single NW*	H_2_S	1 ppm (120 °C)	48 s/56 s	[54]

ZnO	nanorods	H_2_	500 ppm (25 °C)	10min/N/A	[55]
		H_2_S	50 ppb (RT, S∼1.7)	N/A	[56]
		Ethanol	1 ppb (300 °C, S∼10)	N/A	[57]
		Methanol	50 ppm (300, S∼3.2)	N/A	[58]
		Ethanol	100 ppm (325 °C, S∼20)	N/A	[59]

	Single NW	H_2_	200 ppm (RT, S∼0.04)	30 s/50–90 s	[60]

WO_3_ nanowires		H_2_S	1ppm (250 °C, S = 48)	N/A	[61]
		NH_3_	10 ppb (room temperature)	N/A	[62]

TeO_2_ nanowires		NO_2_	10 ppm (26 °C)	10 min	[63]
		NH_3_	10 ppm (26 °C)	>30 min	
		H_2_S	50 ppm (26 °C)	N/A	

CuO	nanowires	CO	30 ppm (300 °C, S∼0.07)	N/A	[64]
		NO_2_	2 ppm (300 °C, S∼0.15)	N/A	

	nanoribbons	Methanol	5 ppm (S∼1.4)	2–4 s/3–7 s	[65]
		Ethanol	5 ppm (200 °C, S∼1.2)	3–6 s/4–9 s	

CdO nanowires		NO_2_	1 ppm (100 °C, S∼0.27)	N/A	[66]

* N/A means not available, NW means nanowire, RT means room temperature.

**Table 2.** Gas sensing properties of metal oxide nanostructure special morphology.

**Material**	**Gas species**	**Sensitivity**	**Response/recovery time**	**Reference**
ZnO brushes	Ethanol	3 (5 ppm)	<10 s/<10 s (10 ppm)	[67]
SnO_2_ brushes	Ethanol	2.3 (0.5 ppm)	4 s	[26]
ZnO dendrites	H_2_S	3.3 (10 ppm)	15–20 s/30–50 s	[68]
ZnO nano-flowers	Ethanol	4.1 (1 ppm)	1–2 s/1–2 s	[69]

**Table 3.** The lowest detection concentrations of various gas species measured of metal oxide nanowires gas sensors.

**Target species**	**Lowest detection concentration Working temperature**	**Materials**	**Reference**
CO	100 ppb, 300 °C	SnO_2_ single nanowire	[70]
NO_2_	1 ppb (estimated, confidence level 3)	V_2_O_5_ nanofibers	[31]
NH_3_	100 ppb, 300 °C	SnO_2_ single nanowire	[40]
Ethanol	100 ppb, 330 °C	SnO_2_ nanofibers	[9]
H_2_	10 ppm (S∼0.4), 300 °C	SnO_2_ nanowires	[45]
H_2_S	50 ppb	ZnO nanorods	[53]

**Table 4.** Metal oxide heterostructured nanostructures gas sensors

**Material**	**Gas Species**	**Sensitivity**	**Response/Recovery Time**	**Ref.**
CuO-SnO_2_ core/shell PN-junction	H_2_S	9.4 × 10^6^ (10 ppm, 60 °C)	N/A	[82]
carbon nanotubes/SnO_2_ core/shell nanostructures	Ethanol	24.5 (50 ppm, room temperature)	1 s/10 s	[83]
α-Fe_2_O_3_/SnO_2_ core-shell nanorods	Ethanol	19.6 (10 ppm, 220 °C)	<30 s/<30 s	[84]
La_2_O_3_ functionalized SnO_2_ nanowires	Ethanol	57.3 (100 ppm, 400 °C)	1 s/110 s	[85]
	Acetone	34.9 (100 ppm, 400 °C)		
Fe_2_O_3_/ZnO core-shell nanorods	90# petroleum	2.73 (5 ppm, 320 °C)	<20 s/<20 s	[86]
	Cyclohexane	1.5 (5 ppm, 320 °C)		
	Ethanol	4.01(5 ppm, 200 °C)		
	Acetone	3.53 (5 ppm, 200 °C)		
SnO_2_ functionalized ZnO nanowires	CO	4.6 (300 ppm, 350 °C)	52 s/550 s	[87]

## References

[1] Yamazoe N. (1991). New approaches for improving semiconductor gas sensors. Sens. Actuat. B.

[2] Comini E., Baratto C., Faglia G., Ferroni M., Vomiero A., Sberveglieri G. (2009). Quasi-one dimensional metal oxide semiconductors: Preparation, characterization and application as chemical sensors. Prog. Mater. Sci..

[3] Pan Z.W., Dai Z.R., Wang Z.L. (2001). Nanobelts of semiconducting oxides. Science.

[4] Morales A., Lieber C.A. (1998). laser ablation method for the synthesis of crystalline semiconductor nanowires. Science.

[5] Comini E., Faglia G., Sberveglieri G. (2002). Stable and highly sensitive gas sensors based on semiconducting oxide nanobelts. Appl. Phys. Lett..

[6] Law M., Kind H., Messer B., Kim F., Yang P.D. (2002). Photochemical sensing of NO_2_ with SnO_2_ nanoribbon nanosensors at room temperature. Angew. Chem. Int. Ed..

[7] Wan Q., Li Q.H., Chen Y.J., Wang T.H., He X.L., Li J.P., Lin C.L. (2004). Fabrication and ethanol sensing characteristics of ZnO nanowire gas sensors. Appl. Phys. Lett..

[8] Raible I, Burghard M, Schlecht, Yasuda U.A., Vossmeyer T. (2005). V_2_O_5_ nanofibres: novel gas sensors with extremely high sensitivity and selectivity to amines. Sens. Actuat. B..

[9] Zhang Y., He X.L., Li J.P., Miao Z.J., Huang F. (2008). Fabrication and ethanol-sensing properties of micro gas sensor based on electrospun SnO_2_ nanofibers. Sens. Actuat. B..

[10] Wang Y, Ramos I., Santiago-Aviles J. (2007). Electrical characterization of a single electrospun porous SnO_2_ nanoribbon in ambient air. Nanotechnology.

[11] Wang Y, Ramos I., Santiago-Aviles J. (2007). Detection of moisture and methanol gas using a single electrospun tin oxide nanofiber. IEEE Sens. J..

[12] Wang G., Ji Y., Huang X.R., Yang X.Q., Gouma P.I., Dudley M. (2006). Fabrication and characterization of polycrystalline WO_3_ nanofibers and their application for ammonia sensing. J. Phys. Chem. B.

[13] Hao R, Yuan J.Y, Peng Q. (2006). Fabrication and sensing behavior of Cr_2_O_3_ nanofibers via in situ gelation and electrospinning. Chem. Lett..

[14] Leon N., Figueroa G., Wang Y, Ramos I., Furlan R., Pinto N., Santiago-Aviles J. (2005). Electrospun tin oxide nanofibers. Nanotechnology II.

[15] Lau M., Dai L., Bosnick K., Evoy S. (2009). Synthesis and characterization of TiOx nanowires using a novel silicon oxide support layer. Nanotechnology.

[16] Qi Q., Zhang T., Yu Q.J., Wang R., Zeng Y., Liu L., Yang H.B. (2008). Properties of humidity sensing ZnO nanorods-base sensor fabricated by screen-printing. Sens. Actuat. B..

[17] Hsueh T.J., Chen Y.W., Chang S.J., Wang S.F., Hsu C.L., Lin Y.R., Lin T.S., Chen I.C. (2007). ZnO nanowire-based CO sensors prepared on patterned ZnO: Ga/SiO2/Si templates. Sens. Actuat. B..

[18] Choi Y.J., Hwang I.S., Park J.G., Choi K.J., Park J.H., Lee J.H. (2008). Novel fabrication of an SnO_2_ nanowire gas sensor with high sensitivity. Nanotechnology.

[19] Hsueh T.J., Hsu C.L., Chang S.J., Chen I.C. (2007). Laterally grown ZnO nanowire ethanol gas sensors. Sens. Actuat. B..

[20] Jung T.H., Kwon S.I., Park J.H., Lim D.G., Choi Y.J., Park J.G. (2008). SnO2 nanowires bridged across trenched electrodes and their gas-sensing characteristics. Appl. Phys. A-Mater. Sci. Proc..

[21] Liao L., Lu H.B., Li J.C., He H., Wang D.F., Fu. D.J., Liu C. (2007). Size dependence of gas sensitivity of ZnO nanorods. J. Phys. Chem. C.

[22] Chen J.J., Wang K., Hartman L., Zhou W.L. (2008). H_2_S Detection by Vertically Aligned CuO Nanowire Array Sensors. J. Phys. Chem. C.

[23] Francioso L., Taurino A.M., Forleo A., Siciliano P. (2008). TiO_2_ nanowires array fabrication and gas sensing properties. Sens. Actuat. B..

[24] Ra Y.W., Choi K.S., Kim J.H., Hahn Y.B., Im H.Y. (2008). Fabrication of ZnO nanowires using nanoscale spacer lithography for gas sensors. Small.

[25] Son J.Y., Lim S.J., Cho J.H., Kim H.J. (2008). Synthesis of horizontally aligned ZnO nanowires localized at terrace edges and application for high sensitivity gas sensor. Appl. Phys. Lett..

[26] Wan Q., Huang J., Xie Z., Wang T.H., Dattoli E.N., Lu W. (2008). Branched SnO2 nanowires on metallic nanowire backbones for ethanol sensors application. Appl. Phys. Lett..

[27] Andrei P., Fields L.L, Zheng J.P., Cheng Y., Xiong P. (2007). Modeling and simulation of single nanobelt SnO_2_ gas sensors with FET structure. Sens. Actuat. B..

[28] Zhang D.H., Li C., Liu X.L., Kasai J., Mozume T., Ishikawa H. (2003). Doping dependent NH_3_ sensing of indium oxide nanowires. Appl. Phys. Lett..

[29] Xu J.H., Wu N.Q., Jiang C.B., Zhao M.H., Li J., Wei Y.G., Mao S.X. (2006). Impedance characterization of ZnO nanobett/Pd Schottky contacts in ammonia. Small.

[30] Zhang N., Yu K., Li L.J., Zhu Z.Q. (2008). Investigation of electrical and ammonia sensing characteristics of Schottky barrier diode based on a single ultra-long ZnO nanorod. Appl. Surf. Sci..

[31] Kim I.D., Rothschild A., Lee B.H., Kim D.Y., Jo S.M., Tuller H.L. (2006). Ultrasensitive Chemiresistors Based on Electrospun Ti_O2_ Nanofibers. Nano Lett..

[32] Ahn M.W., Park K.S., Heo J.H., Park J.G., Kim D.W., Choi K.J., Lee J.H., Hong S.H. (2008). Gas sensing properties of defect-controlled ZnO-nanowire gas sensor. Appl. Phys. Lett..

[33] Zhang Y., Kolmakov A., Chretien S., Metiu H., Moskovits M. (2004). Control of Catalytic Reactions at the Surface of a Metal Oxide Nanowire by manipulating electron density inside it. Nano Lett..

[34] Maiti A., Rodriguez J.A., Law M., Kung P., McKinney J.R., Yang P.D. (2003). SnO_2_ Nanoribbons as NO_2_ Sensors: Insights from first principles calculations. Nano Lett..

[35] Feng P., Wan Q., Wang T.H. (2005). Contact-controlled sensing properties of flowerlike ZnO nanostructures. Appl. Phys. Lett..

[36] Zhang D.H., Liu Z.Q., Li C., Tang Tao, Liu X.L., Han S., Lei B., Zhou C.W. (2004). Detection of NO_2_ down to ppb levels using individual and multiple In_2_O_3_ nanowire devices. Nano Lett..

[37] Rout C.S., Govindaraj A., Rao C.N.R. (2006). High-sensitivity hydrocarbon sensors based on tungsten oxide nanowires. J. Mater. Chem..

[38] Li C., Zhang D.H., Liu X.L., Han S., Tang T., Han J., Zhou C.W. (2003). In2O3 nanowires as chemical sensors. Appl. Phys. Lett..

[39] Zhang Y., He XL., Li J.P., Miao Z.J., Huang F. (2008). Fabrication and ethanol-sensing properties of micro gas sensor based on electrospun SnO_2_ nanofibers. Sens. Actuat. B.

[40] Meier D.C., Semancik S., Button B., Strelcov E., Kolmakova A. (2007). Coupling nanowire chemiresistors with MEMS microhotplate gas sensing platforms. Appl. Phys. Lett..

[41] Chen Y.Q., Cui X.F., Zhang K., Pan D.Y., Zhang S.Y., Wang B., Hou J.G. (2003). Bulk-quantity synthesis and self-catalytic VLS growth of SnO_2_ nanowires by lower-temperature evaporation. Chem. Phys. Lett..

[42] Chen P.C., Shen G.Z., Zhou C.W. (2008). Chemical Sensors and Electronic Noses Based on 1-D Metal Oxide Nanostructures. IEEE Trans. Nanotech..

[43] Sysoev V.V., Button B.K., Wepsiec K., Dmitriev S., Kolmakov A. (2006). Toward the Nanoscopic “Electronic Nose”: Hydrogen vs Carbon Monoxide Discrimination with an Array of Individual Metal Oxide Nano- and Mesowire Sensors. Nano Lett..

[44] Hernandez-Ramirez F., Barth S., Tarancon A., Casals O., Pellicer E., Rodriguez J., Rodriguez A.R., Morante J.R., Mathur S. (2007). Water vapor detection with individual tin oxide nanowires. Nanotechnology.

[45] Ying Z., Wan Q., Song Z.T. (2004). SnO_2_ nanowhiskers and their ethanol sensing characteristics. Nanotechnology.

[46] Wang B., Zhu L.F., Yang Y.H. (2008). Fabrication of a SnO2 nanowire gas sensor and sensor performance for hydrogen. J. Phys. Chem. C.

[47] Wang B., Zhu L.F., Yang Y.H., Xu N.S., Yang G.W. (2008). Fabrication of a SnO2 nanowire gas sensor and sensor performance for hydrogen. J. Phys. Chem. C.

[48] Huang H., Lee Y.C., Tan O.K. (2009). High sensitivity SnO_2_ single-nanorod sensors for the detection of H_2_ gas at low temperature. Nanotechnology.

[49] Kuang Q., Lao C.S., Wang Z.L., Xie Z.X., Zheng L.S. (2007). High-sensitivity humidity sensor based on a single SnO_2_ nanowire. J. Am. Chem. Soc..

[50] Chu X.F., Wang C.H., Jiang D.L., Chen M.Z. (2004). Ethanol sensor based on indium oxide nanowires prepared by carbothermal reduction reaction. Chem. Phys. Lett..

[51] Kaur M., Jain N., Sharma K., Bhattacharya S., Roy M., Tyagi A.K., Gupta S.K., Yakhmi J.V. (2008). Room-temperature H_2_S gas sensing at ppb level by single crystal In_2_O_3_ whiskers. Sens. Actuat. B..

[52] Xu P.C., Cheng Z.X., Pan Q.Y., Xu J.Q., Xiang Q., Yu W.J., Chu Y.L. (2008). High aspect ratio In_2_O_3_ nanowires: Synthesis, mechanism and NO_2_ gas-sensing properties. Sens. Actuat. B..

[53] Zeng Z.M., Wang K., Zhang Z.X., Chen J.J., Zhou W.L. (2009). The detection of H_2_S at room temperature by using individual indium oxide nanowire transistors. Nanotechnology.

[54] Xu J.Q., Chen Y.P., Shen J.N. (2008). Ethanol sensor based on hexagonal indium oxide nanorods prepared by solvothermal methods. Mater. Lett..

[55] Wang H.T., Kang B.S., Ren F., Tien L.C., Sadik P.W., Norton D.P., Pearton S.J., Lin J.S. (2005). Hydrogen-selective sensing at room temperature with ZnO nanorods. Appl. Phys. Lett..

[56] Wang C.H., Chu X.F., Wu M.W. (2006). Detection of H_2_S down to ppb levels at room temperature using sensors based on ZnO nanorods. Sens. Actuat. B..

[57] Yang Z., Li L.M., Wan Q. (2008). High-performance ethanol sensing based on an aligned assembly of ZnO nanorods. Sens. Actuators B.

[58] Cao Y.L., Hu P.F., Pan W.Y., Huang Y.D., Jia D.Z. (2008). Methanal and xylene sensors based on ZnO nanoparticles and nanorods prepared by room-temperature solid-state chemical reaction. Sens. Actuat. B.

[59] Ge C.Q., Bai Z.K., Hu M.L. (2008). Preparation and gas-sensing property of ZnO nanorod-bundle thin films. Mater. Lett..

[60] Lupan O., Chai G, Chow L. (2008). Novel hydrogen gas sensor based on single ZnO nanorod. Microelectr. Eng..

[61] Rout C.S., Hegde M., Rao C.N.R. (2008). H_2_S sensors based on tungsten oxide nanostructures. Sens. Actuat. B.

[62] Zhao Y.M., Zhu Y.Q. (2009). Room temperature ammonia sensing properties of W_18_O_49_ nanowires. Sens. Actuat. B.

[63] Liu Z.F., Yamazaki T., Shen Y. (2007). Room temperature gas sensing of p-type TeO_2_ nanowires. Appl. Phys. Lett..

[64] Kim Y.S., Hwang I.S., Kim S.J. (2008). CuO nanowire gas sensors for air quality control in automotive cabin. Sens. Actuat. B.

[65] Gou X.L., Wang G.X., Yang J.S., Park J., Wexler D. (2008). Chemical synthesis. characterisation and gas sensing performance of copper oxide nanoribbons. J. Mater. Chem..

[66] Guo Z., Li M.L., Liu J.H. (2008). Highly porous CdO nanowires: preparation based on hydroxy- and carbonate-containing cadmium compound precursor nanowires, gas sensing and optical properties. Nanotechnology.

[67] Zhang Y., Xu J.Q., Xiang Q., Li H., Pan Q.Y., Xu P.C. (2009). Brush-Like Hierarchical ZnO Nanostructures: Synthesis, photoluminescence and gas sensor properties. J. Phys. Chem. C.

[68] Zhang N., Yu K., Li Q., Wan Q. (2008). Room-temperature high-sensitivity H_2_S gas sensor based on dendritic ZnO nanostructures with macroscale in appearance. J. Appl. Phys..

[69] Li C.C., Du Z.F., Li L.M., Yu H.C., Wan Q., Wang T.H. (2007). Surface-depletion controlled gas sensing of ZnO nanorods grown at room temperature. Appl. Phys. Lett..

[70] Ramírez F.H., Tarancón A., Casals O., Arbiol AJ., Rodríguez R., Morante J.R. (2007). High response and stability in CO and humidity measures using a single SnO_2_ nanowire. Sens. Actuat. B..

[71] Arbiol J., Cirera A., Peiró F., Cornet A., Morante J.R., Delgado J.J., Calvino J.J. (2002). Optimization of tin oxide nanosticks faceting for the improvement of palladium nanoclusters epitaxy. Appl. Phys.Lett..

[72] Kolmakov A., Klenov D.O., Lilach Y., Stemmer S., Moskovits M. (2005). Enhanced gas sensing by individual SnO2 nanowires and nanobelts functionalized with Pd catalyst particles. Nano Lett..

[73] Shen Y.B., Yamazaki T., Liu Z.F., Meng D., Kikuta T., Nakatani N., Saito M., Mori M. (2009). Microstructure and H_2_ gas sensing properties of undoped and Pd-doped SnO_2_ nanowires. Sens. Actuat. B..

[74] Wang H.T., Kang B.S., Ren F., Tien L.C., Sadik P.W., Norton D.P., Pearton S.J., Lin J.S. (2005). Hydrogen-selective sensing at room temperature with ZnO nanorods. Appl. Phys. Lett..

[75] Chang S.J., Hsueh T.J., Chen I.C., Hsieh S.F., Chang S.P., Hsu C.L., Lin Y.R., Huang B.R. (2008). Highly sensitive ZnO nanowire acetone vapor sensor with Au adsorption. IEEE Trans. Nanotechnol.

[76] Hsueh T.J., Chang S.J., Hsu C.L., Lin Y.R., Chen I.C. (2007). Highly sensitive ZnO nanowire ethanol sensor with Pd adsorption. Appl. Phys. Lett..

[77] Chang S.J., Hsueh T.J., Chen I.C., Huang B.R. (2008). Highly sensitive ZnO nanowire CO sensors with the adsorption of Au nanoparticles. Nanotechnology.

[78] Shen Y.B., Yamazaki T., Liu Z.F., Meng D., Kikuta T., Nakatani N., Saito M., Mori M. (2009). Microstructure and H_2_ gas sensing properties of undoped and Pd-doped SnO_2_ nanowires. Sens. Actuat. B.

[79] Liao L., Mai H.X., Yuan Q., Lu H.B., Li J.C., Liu C., Yan C.H., Shen Z.X., Yu T. (2008). Single CeO_2_ nanowire gas sensor supported with Pt nanocrystals: Gas sensitivity, surface bond states, and chemical mechanism. J. Phys. Chem. C.

[80] Ramgir N.S., Mulla I.S., Vijayamohanan K.P. (2005). A room temperature nitric oxide sensor actualized from Ru-doped SnO_2_ nanowires. Sens. Actuat. B.

[81] Wan Q., Wang T.H. (2005). Single-crystalline Sb-doped SnO2 nanowires: synthesis and gas sensor application. Chem. Commun..

[82] Xue X.Y., Xing L.L., Chen Y.J., Shi S.L., Wang Y.G., Wang T.H. (2008). Synthesis and H_2_S sensing properties of CuO-SnO_2_ core/shell PN-junction nanorods. J. Phys. Chem. C.

[83] Chen Y.J., Zhu C.L., Wang T.H. (2006). The enhanced ethanol sensing properties of multi-walled carbon nanotubes/SnO_2_ core/shell nanostructures. Nanotechnology.

[84] Chen Y.J., Zhu C.L., Wang L.J., Gao P., Cao M.S., Shi X.L. (2009). Synthesis and enhanced ethanol sensing characteristics of alpha-F_e2O3_/Sn_O2_ core-shell nanorods. Nanotechnology.

[85] Si S.F., Li C.H., Wang X., Peng Q., Li Y.D. (2006). Fe_2_O_3_/ZnO core-shell nanorods for gas sensors. Sens. Actuat. B.

[86] Wang J.X., Sun X.W., Xie S.S., Yang Y., Chen H.Y., Lo G.Q., Kwong D.L. (2007). Preferential growth of SnO_2_ triangular nanoparticles on ZnO nanobelts. J. Phys. Chem. C.

[87] Van N.H., Kim H.R, Ju B.K., Lee J.H. (2008). Enhanced performance of SnO_2_ nanowires ethanol sensor by functionalizing with La_2_O_3_. Sens. Actuat. B.

[88] Chen P.C., Ishikawa F.N., Chang H.K., Ryu K., Zhou C.W. (2009). A nanoelectronic nose: a hybrid nanowire/carbon nanotube sensor array with integrated micromachined hotplates for sensitive gas discrimination. Nanotechnology.

[89] Goschni J. (2001). An electronic nose for intelligent consumer products based on a gas analytical gradient microarray. Microelectron. Engineer..

[90] Sysoev V.V., Goschnick J., Schneider T. (2007). A gradient microarray electronic nose based on percolating SnO_2_ nanowire sensing elements. Nano Lett..

[91] Strelcov E, Dmitriev S., Button B., Cothren J., Sysoev V., Kolmakov A. (2008). Evidence of the self-heating effect on surface reactivity and gas sensing of metal oxide nanowire chemiresistors. Nanotechnology.

[92] Prades J.D., Jimenez R.D., Hernandez F.R., Barth S., Cirera A., Rodriguez A.R., Mathur S., Morante J.R. (2008). Ultralow power consumption gas sensors based on self-heated individual nanowires. Appl. Phys. Lett..

[93] Faglia G., Baratto C., Sberveglieri G., Zha M., Zappettini A. (2005). Adsorption effects of NO_2_ at ppm level on visible photoluminescence response of SnO_2_ nanobelts. Appl. Phys. Lett..

[94] Lettieri S., Bismuto A., Maddalena P., Baratto C., Comini E., Faglia G., Sberveglieri G., Zanotti L. (2006). Gas sensitive light emission properties of tin oxide and zinc oxide nanobelts. J. Non-Cryst. Solids.

[95] Comini E., Baratto C., Faglia G., Ferroni M., Sberveglieri G. (2007). Single crystal ZnO nanowires as optical and conductometric chemical sensor. J. Phys. D.

[96] Setaro A., Bismuto A., Lettieri S., Maddalena P., Comini E., Bianchi S., Baratto C., Sberveglieri G. (2008). Optical sensing of NO_2_ in tin oxide nanowires at sub-ppm level. Sens. Actuat. B.

[97] Zhang X.J., Wang G.F., Zhang W., Hu N.J., Wu H.Q., Fang B. (2008). Seed-mediated growth method for epitaxial array of CuO nanowires on surface of Cu nanostructures and its application as a glucose sensor. J. Phys. Chem. C.

[98] Ansari S.G., Wahab R., Ansari Z.A., Kim Y.S., Khang G., Hajry A.A., Shin H.S. (2009). Effect of nanostructure on the urea sensing properties of sol–gel synthesized ZnO. Sens. Actuat. B.

[99] Zhang X.J., Wang G.F., Wang Q., Zhao L.J., Wang M., Fang B. (2009). Cupreous oxide nanobelts as detector for determination of l-Tyrosine. Mater. Sci. Eng. B.

[100] Fenster C., Smith A.J., Abts A., Milenkovic S., Hassel A.W. (2008). Single tungsten nanowires as pH sensitive electrodes. Electro. Commun..

